# A large abdominal cyst as a manifestation of pelviureteric junction obstruction in a neonate

**DOI:** 10.12669/pjms.40.2(ICON).8980

**Published:** 2024-01

**Authors:** Syed Faisal Usman, Athar Razaq, Ejaz Ahmad, Abdul Rehman Rashid, Maham Shehzadi

**Affiliations:** 1 Syed Faisal Usman, FCPS Consultant, Department of Pediatric Surgery, Recep Tayyip Erdoğan Hospital (RTEH), Muzaffargarh, Punjab, Pakistan; 2 Athar Razaq, FCPS Consultant, Department of Neonatology, Recep Tayyip Erdoğan Hospital (RTEH), Muzaffargarh, Punjab, Pakistan; 3 Ejaz Ahmad, FCPS Consultant, Department of Neonatology, Recep Tayyip Erdoğan Hospital (RTEH), Muzaffargarh, Punjab, Pakistan; 4 Abdul Rehman Rashid, FCPS Registrar, Department of Pediatric Surgery, Recep Tayyip Erdoğan Hospital (RTEH), Muzaffargarh, Punjab, Pakistan; 5 Maham Shehzadi, MBBS. Final Year Resident Pediatrics, Department of Neonatology, Recep Tayyip Erdoğan Hospital (RTEH), Muzaffargarh, Punjab, Pakistan

**Keywords:** Intraabdominal cystic lesions, Neonatal intensive care unit (NICU), Pelviureteric junction obstruction (PUJO), Percutaneous nephrostomy (PCN), Ultrasound (USG)

## Abstract

Intraabdominal cystic lesions diagnosed during antenatal period are uncommon. They are found to have varying origins, with renal tract being the most common site. Rarely, a large unilateral cystic lesion of renal origin is caused by Pelviureteric junction obstruction, crossing the midline, leading to compression of the contralateral kidney. We present a case of a neonate who was diagnosed with a large abdominal cyst in the antenatal period. The cyst persisted and crossed the midline causing hydronephrosis on the contralateral side. This is an unusual presentation of a commonly occurring condition, usually such large cyst at birth origins from alimentary tract rather renal system. It is important to understand unusual presentations of intraabdominal lesions and the associated pathology. It is mandatory to rule out renal obstruction, if there is any decompression of renal function, it is mandatory to save renal function till the time of definitive surgery.

## INTRODUCTION

Antenatally detected abdominal masses are usually cystic in nature.^1^ Among neonates, approximately one is affected in 1,500 live births.^2^ Abdominal cystic lesions may have different origins e.g., mesenteric or omental cysts, intestinal duplication cysts, hepatic cyst, ovary or renal, and adrenal cysts etc.^3^ The majority of antenatally diagnosed abdominal cystic lesions originate from renal tract.^4^ In neonates, the main cause of unilateral mass of renal origin is Pelviureteric junction obstruction.^5^ It is rare that a Pelviureteric junction obstruction becomes so severe that it results in a large abdominal cyst without recognizable surrounding parenchyma and puts compressive effects on the opposite kidney.^4^ Here, we present a case of a newborn who was antenatally identified with a large abdominal cyst of unknown origin and postnatally diagnosed with left large abdominal cyst along with pelviureteric junction obstruction crossing the midline and exerting compressive effects on the contralateral right kidney.

## CASE REPORT

A known hypertensive 23-year-old mother, G3 P0 A2 regularly attending antenatal clinics, gave birth to a 35 week baby boy through C-section. The neonatal resuscitation team was informed of the birth and the newborn was attended in the resuscitation room. Birth weight was 3.5 kg. It was an uneventful delivery, cry was immediate, and his Apgar score was eight and nine at one and five minutes respectively. On last antenatal scan at 34 weeks of gestation, AFI was 22 cm. USG revealed only right kidney and a large abdominal cyst of with no obvious origin.

He was shifted from the resuscitation room to neonatal intensive care unit (NICU) for further evaluation. While being cared for in NICU on an open warmer, abdominal distension was noted with no other obvious physical examination findings. Abdomen was distended with central umbilicus, no prominent veins, erythema or visible peristalsis. Abdomen was soft and a lump/mass of approximately 8.5 cm was felt in the left lumbar area. Anticipating his antenatal diagnosis, postnatal USG abdomen was planned. It showed the right kidney only measuring 4.5 x 2.3 x 0.4 cm and a left sided large cystic lesion measuring 88 x 50 x 81 mm with compression effects over distal and mid right ureter causing moderate right sided hydronephrosis measuring 20mm in AP diameter. Urinary bladder was also visualized.

**Fig.1:**
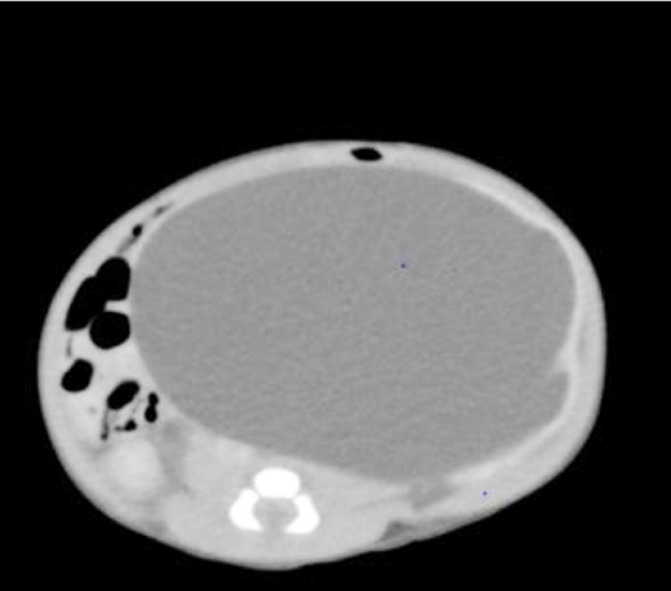
CT scan of Abdomen.

As per protocol of pediatric urology, antibiotics cover was initiated. He had high blood pressure, at 99 percentile and hence was started on antihypertensive, amlodipine. He was reluctant to feed and was lethargic. Considering his condition and suspecting hypertensive encephalopathy, extracted breast milk was given through nasogastric tube and base line blood tests were requested. CT abdomen was performed which showed left gross hydronephrosis measuring 79 x 107 x 83 mm with marked cortical thinning and Pelviureteric junction (PUJ) narrowing. Gross finding showed crossing the midline and causing compressing effects on right ureter resulting in moderate hydronephrosis measuring 20 x 15 mm, shown in Fig.1.

**Fig.2.1:**
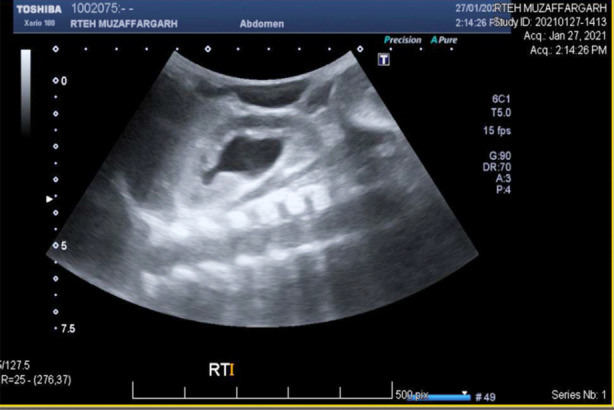
Ultrasound Scan.

**Fig.2.2:**
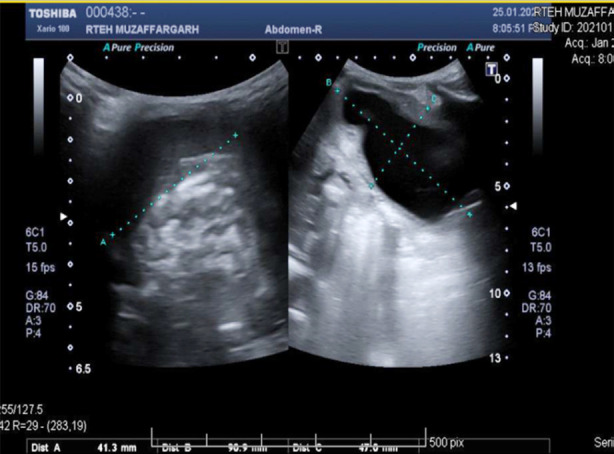
Ultrasound Scan.

Patient had consultation with both nephrologist and radiologist. According to the plan percutaneous nephrostomy (PCN) was done and the interventional radiologist placed a left PCN catheter. Post procedure USG revealed a well sited left PCN in place with mild right hydronephrosis. USG findings shown Fig.2.1 and Fig.2.2.

**Fig.3:**
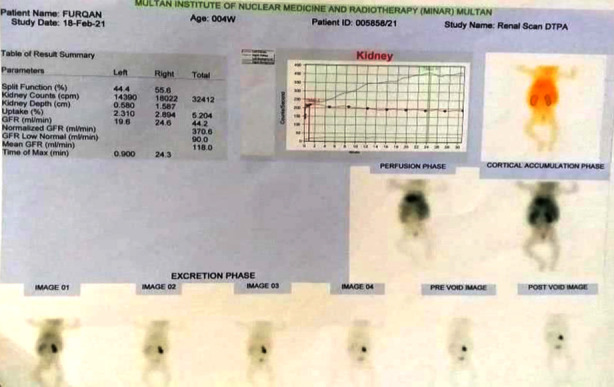
99m Tc-DTPA Renal Scan.

Patient was then followed up in the outpatient clinic (OPD). To evaluate bilateral (B/L) kidney function status, Tc-DTPA Scan was done that showed B/L reduced functioning kidneys with evidence of partial outflow obstruction, shown in Fig.3.

**Fig.4:**
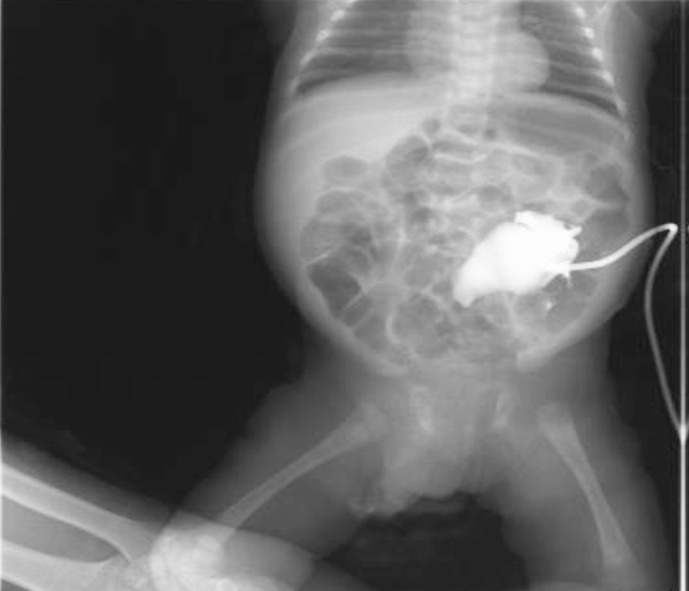
Antegrade Nephrostogram.

An anterograde nephrostogram was also done to investigate the obstruction in detail. The nephrostogram image is shown in Fig.4. A Pyeloplasty with double J stent placement was planned and done to relieve the obstruction. After six weeks, the stent was removed and the patient is on regular OPD follow up.

## DISCUSSION

Congenital abdominal cystic lesions (CACL) are defined as non-solid lesions located in the abdominal cavity. It is due to better radiological approach and experience during standard prenatal sonographic screening of newborns that the diagnostic rate is increasing.^2^ Among the differential diagnosis of cystic structures the list includes either originating from the gastrointestinal tract e.g. mesenteric or omental cysts, intestinal duplication cysts, hepatic or choledochal cysts etc. or from the genitourinary tract e.g. ovarian, renal, urachal, and adrenal cysts.^3^

The diagnosis can be aided by keeping in mind the exact location of cyst, gender of neonate and the prevalence of different cysts in abdomen. Most common origin of abdominal cysts is from the renal tract which include hydronephrosis, multicystic dysplastic kidney, ureteric dilatation and dilatation of urinary bladder etc.^4^ It accounts for 30% to 50% of urinary tract anomalies in newborns.^6^ Among renal anomalies, the main cause of unilateral hydronephrosis is pelviureteric junction obstruction (PUJO), with an incidence of about one in 1000 to 1500 newborns. Its pathophysiological causes are divided into intrinsic and extrinsic. The intrinsic obstruction though unclear, is considered to be caused by muscular discontinuity at the site of the PUJ, improper innervation, and ureteral hypoplasia. The main cause behind extrinsic obstruction is compression from an overlying renal vessel and it is diagnosed later as compared to intrinsic obstruction.^5^ Although rarely, it has been seen that the PUJ obstruction is significantly causing a large abdominal cyst with unrecognizable surrounding parenchyma.^4^ A massive urinary tract cyst crossing the midline and causing hydronephrosis of the contralateral kidney is quite rare.

In the assessment protocol after physical examination, USG is done as the foremost modality when suspecting a gross cystic lesion or hydronephrosis to quantify risk and determine the degree of evaluation. The timing of the USG depends upon the degree of suspected obstruction. Mild to moderate hydronephrosis are evaluated within three to seven days. Severe hydronephrosis must be evaluated early within 24 to 48 hours post-delivery. Subsequently radionuclide renal scan is done to assess renal function and degree of obstruction.^5^ Tc- MAG3 scan is preferably used in newborns at age of 6 weeks and it is more accurate assessment of function and drainage than Tc-DTPA. Tc-MAG3 scan is mostly preferred modality if associated structural anomaly is present.^7^ The management can be tailored, depending upon the degree of obstruction and renal function of the affected kidney.^8^ Literature suggests many ways of management of PUJO in neonates but limited details are available on gross hydronephrosis. If renal functioning is impaired by 5-20%, PCN and ultimately Pyeloplasty is done. Pyeloplasty is recommended for 20-40% renal function. If patient condition is not apt for other treatment options, then drainage procedures may provide relief.^1^

In some retrospective data it has been shown that surgical intervention rates are as high as 55% in patients with conservative management. However, a prospective randomized study done by SFU while comparing conservative management and surgical approach showed 25% rate of surgical interventions in the conservative management group. However, in either case, renal function stabilization was similar.^5^

## CONCLUSION

It is important to understand the pathology and unusual presentations. It is always mandatory to rule out renal obstruction, if there is any decompression of renal function, it is mandatory to save renal function till the time of definitive surgery. As, this child was hypertensive, antihypertensive treatment included with the case management with the help of our neonatology team.

### Authors’ Contribution:

**SFU:** Prepared the reports with all necessary data and is responsible for accuracy and integrity of the report.

**AR:** helped in literature review.

**EA:** Helped in writing discussion.

**AR:** Helped in setting tables and images.

**MS:** Helped in proof reading and review.
